# Medico-Topographical Remarks on the County of Fauquier, Va.

**Published:** 1853-06

**Authors:** G. R. B. Horner

**Affiliations:** Surgeon U. S. Naval Station, Philadelphia


### *Medico-Topographical Remarks on the County of Fauquier, Va.*

By G. R. B. Horner, M. D., Surgeon U. S. Naval Station, Philadelphia.

This county—one of the largest in the State—lies forty miles westward of the District of Columbia, and extends fifty miles from North to South. On the East it is bounded by Broad Run, one of the tributary streams of the Potomac; and on the South-west by the Hedgmon, the northern branch of the Rappahannock. The lower half of the county is gently undulated and level, the upper portion generally hilly and mountainous. The soil of the former is very sandy in parts,—alluvial and clayey in others. Hence its marshyness and unwholesomeness during wet weather. The soil of the latter, or northern portion, is generally loamy, lighter, and more productive; but there are exceptions, as the part termed Marshall's manor, in the western portion of the county, which is glady, sandy, and barren to a great extent. Other exceptions might be made; but the soil of the northern portion may be justly called fertile, both naturally and artificially. Grain of every kind, clover, hay, various fruits, and vegetables abound; farms are in fine order, and sell at high rates. The most valuable mineral production is gold, which is found in the white flint or quartz rocks, about the central part of the county, in small quantities, but more abundantly in the mines at the southern part. Slate, black rock, limestone, sandstone of fine quality,—for paving and building,—are found plentifully in quarries here and there; but limestone chiefly north of the Rappahannock mountain. In the sandy parts the pine flourishes; in the flinty the chestnut; in other parts grow the red, white and black oak, the walnut, white poplar, cornus florida, hickory, and other forest trees. Interspersed are the haw, quince, chinquepin, cedar, locust, cherry, apple, pear, peach, persimmon; and many medicinal plants, as the dewberry, serpentaria, stramonium, taraxicum, night-shades, sage, horehound, green and spear-mint, pennyroyal, hop, wild turnip, chenopodium anthelminticum and sassafras. Some of these plants are due to cultivation, others grow spontaneously and luxuriously, when located in suitable soils—as the green mint along branches; the pennyroyal or mentham pelegium on the road sides, and hills uncovered by forests. In neglected fields the dew and blackberry and luscious strawberry abound, until the young pines shoot up, spontaneously, and ultimately overwhelm them. This occurs with surprising rapidity; and in a few years once barren land is seen shaded by a dense, high waving forest of these trees. In a short time longer, they become fit for fences and buildings, and always afford shelter to wild and domestic animals during winter, and the severe storms of rain, thunder and lightning, so common in summer. Cattle are the most abundant and profitable of the latter. Vast numbers are driven, when half grown, from the north-western part of the State, grazed for a year, and then sold at a large advance,—usually at double price,—from the richness of the pastures, and the abundance of fine water found in every valley.

Hares, opossums, raccoons, foxes, squirrels, larks, robins, woodcock, snipe, sparrows, blackbirds of several species, part ridges, and wild turkeys, are the principal game. Wild pigeons occasionally appear, and are killed in great numbers. Hawks, and especially crows, are the pests of the land, and the turkey-buzzard, in mild weather, is ever seen soaring aloft in quest of his epicurean fare.

From the proximity of the Blue Ridge, which bounds the northern and north-western parts of the county, and from the height of the other mountains, as well as other causes, the climate is cool and variable,—subject to great changes in temperature, and in warm weather to occasional tornadoes of great violence, which always come from towards the mountains. Within a few years, several of the former have occurred, and blown down dwellings and other houses, including a large brick church in Warrenton, the county town—a handsome one, situated on an elegant hill in the centre of Fauquier, 650 feet above tide-water, and overlooking the whole of the lower part of the county. Hail-storms sometimes happen; and one which took place about thirty-five years ago was so violent and destructive to vegetation and buildings, that it cannot be forgotten. It then fell in lumps so large as to shiver every glass struck, to kill grass, and leave its marks not only on it, but on every housetop.

From this vicissitude of climate, pulmonary affections are very common; pleurisy, pneumonia, and phthisis are familiar causes of death; and scarlet fever, in winter, has been still more fatal among children. In 1815 the typhus pneumonia prevailed extensively in the low, damp country south and east of the town, and carried off many victims. Rheumatism abounds among the slaves, and in summer miasmatic fevers, remittent and intermittent, occur in some parts, near mill-dams and large streams; but of late years the bilious fever has been rare. Indeed, from Dr. Chilton, one of the chief physicians in the town, I understand he has hardly ever met with it, and that another physician, of much greater experience, informed him he had not had a case for ten years. Notwithstanding the climate is faulty, the inhabitants of Fauquier are distinguished for health, activity, energy, and enterprise, and attain as great longevity as any in the State. Those who become invalids have access to that delightful and commodious watering-place, called *Lee's*, or the Warrenton Springs. They are on the left bank of the Rappahannock, six miles southwest of the town, and accessible by a well-graded turnpike extending from the town to them. The railroad from Alexandria to it renders them very convenient to Eastern invalids; and as the water is sulphurous, magnesian, and palatable, it is found well adapted to those affected with digestive disorders, and particularly dyspepsia. But of late, an alum spring in the adjoining county of Culpepper has become a rival; and I found a lady, who had long suffered from this complaint, using it largely, having procured a supply at her residence in Warrenton. But the most important case occupying the medical faculty was one of medical jurisprudence. The facts were these, so far as I could learn by attending the trial and from report:—Last fall, two negro men kindled a fire on the eastern branch of the above river, near the springs, and engaged at night in fishing. While there, two overseers—Hunter and Curtis—forming a patrol, and intoxicated, it was asserted, discerned the fire and negroes, and hailed them. In answer, as it was dark, and they could not see distinctly, they inquired whether the former were white or black. This enraged the patrol, and, rushing up, they assaulted the negroes: one fled and saved himself; the other was seized, beaten, thrown down, and choked to death, without his assailants being fully aware of it, as they dragged him to an adjacent house and left him. Anxious concerning him, they returned after a while, and finding him dead, to conceal their guilt, dragged him along a branch, and threw him into the river where it was not over a foot deep. He was there found; an inquest of physicians and others was held, and his body was dissected. From the marks of violence about it, the bruises on the face, engorgement of the lungs, and bloated features, they were satisfied he was strangled. The overseers were apprehended, made some confessions, retracted them, and were tried in April before Judge Tyler of the Superior Court. The counsel for the defence cross-questioned the witnesses, puzzled the doctors no little, and endeavored to prove that the man was either drowned or unintentionally killed; and to sustain him in the first position, urged the fact of some water having been found in the lungs. After hearing the arguments on both sides, the jury—believed to have been packed from persons of a like kind to the criminals—retired, and in a short time returned into court, and rendered a verdict of death "from involuntary homicide." When the learned judge heard this, he became enraged, reprimanded both jury and criminals, and told the first he did not believe any other persons in the courthouse besides themselves believed the deceased owed his death to that cause. He then imposed on the prisoners a heavy fine—with the price of the negro, it was said, amounting to $2000—and sentenced them to three months' longer imprisonment. To end the chapter, it may be well to state that incensed justice or some malignant wretch, on the first day of this month, applied the torch to the courthouse steeple during the night; and that handsome edifice, so many years the pride and admiration of the country, crumbled into ruins, while its antique bell, tumbling from its dizzy height, gave its last knell and sounding its own funeral dirge, sank into the fiery gulf beneath. It was there soon irrecoverably melted. At this sad spectacle every beholder mourned; and among the sorrowful crowd looking on—as the Jews of old in sight of their burning temple—was an old negro man, leaning against the brick enclosure of the courthouse, and sobbing bitterly, that one of his race had so recently been allowed to be murdered, and to remain unavenged. Who was the incendiary is still a conjecture. Many have been suspected—whites and blacks, slaves and freemen, have been accused of burning this and the many other houses burnt in Warrenton within the last two years. A member of the bar has been accused of it, and acquitted. From the burning of the courthouse so soon after the above trial and unsatisfactory sentence of the overseers, reason is entertained of that having been caused by the negro who escaped from them, or some other one offended at their being so slightly punished; but the author of this fire is still unknown, and we shall have to ascribe it to that very common and convenient origin of mischief in modern times, spontaneous combustion, which is supposed to have caused the late destruction of such a great amount of public property at the New York navy-yard. To the same cause we might plausibly ascribe all the fires mentioned before, had they not occurred in the cool of the night, instead of the heat of the day, when there is so much greater tendency to that phenomenon.

